# Changes in the Disparity Vergence Main Sequence after Treatment of Symptomatic Convergence Insufficiency in Children

**DOI:** 10.16910/jemr.12.4.6

**Published:** 2019-12-04

**Authors:** Mitchell Scheiman, Chang Yaramothu, Tara L. Alvarez

**Affiliations:** Pennsylvania College of Optometry at Salus University, Elkins Park, PA, USA; New Jersey Institute of Technology, Newark, NJ, USA

**Keywords:** main sequence, disparity vergence, objective eye movement measurements, convergence insufficiency, office-based vergence and accommodative therapy, vision therapy, near point of convergence, orthoptics

## Abstract

This study investigates the underlying physiological mechanisms that may lead to improved outcomes for symptomatic convergence insufficiency (CI) patients after 12 weeks of office-based vergence/accommodation therapy (OBVAT) by evaluating the change in the main sequence of vergence and saccadic eye movements. In this prospective trial, 12 participants with symptomatic CI were recruited and treated with 12 weeks of OBVAT. Outcome measures included the objective assessment of the following: peak velocity, time to peak velocity, latency, response amplitude, and clinical changes in the near point of convergence (NPC), positive fusional vergence (PFV) and symptoms via the Convergence Insufficiency Symptom Survey (CISS). Ten of the twelve participants (83%) were categorized as “successful” and two were “improved” based on pre-determined published criteria (CISS, NPC, PFV). There were statistically significant changes in peak velocity, time to peak velocity, and response amplitude for both 4° and 6° symmetrical convergence and divergence eye movements. There was a significant change in the main sequence ratio for convergence post-OBVAT compared to baseline measurements (P=0.007) but not for divergence or saccadic responses. Phasic/step vergence movements adjust the underlying neural control of convergence and are critical within a vision therapy program for CI patients.

## Introduction

Convergence insufficiency (CI) is defined as a condition in which there is greater exophoria at near than at distance, a receded near point of convergence, and reduced positive fusional vergence at near [1, 2]. Individuals with this condition may experience symptoms related to near work that interfere with school, work, and leisure activities [3, 4]. The most effective treatment for convergence insufficiency is office-based vergence/ accommodative therapy (OBVAT)[5-9] which is designed to increase the compensatory ability of the visual system (positive fusional vergence) to overcome the tendency for the eyes to drift outward, and to improve the dynamics (velocity, latency, gain) of the disparity vergence response. Several randomized clinical trials have demonstrated that 12 to 16 weekly, one hour vision therapy visits, effectively restore normal clinical function in about 75% of children [7, 8, 10, 11] and adults [5, 9] with symptomatic convergence insufficiency. However, the gap in our knowledge is that we do not completely understand the underlying physiological mechanisms that contribute to the reduction in symptoms and improvement in clinical findings that have been demonstrated in these clinical trials. A better understanding of these mechanisms may help researchers and clinicians develop new therapy protocols that would improve success rates.

Prior pilot studies [12, 13] suggest that the main sequence, a plot of maximum response velocity as a function of response amplitude, may be a useful tool to better understand how OBVAT improves binocular vision function. The original main sequence analysis of saccades was utilized to study the underlying neural control of saccadic responses for a wide range saccadic response magnitudes ranging from about 0.5 deg to about 50 deg [14]. For many studies of vergence eye movements, the magnitude of the response are of a reduced range; hence a main sequence ratio of peak velocity divided by response amplitude has been investigated to isolate and explore the preprogrammed, fusion-initiating component of disparity vergence [13, 15-18].

Jones [19] was the first to describe disparity vergence as having a high velocity, fusion-initiating component (FIC), which is open-loop with respect to the visual stimulus, and a second, slower component, the fusion sustaining component (FSC) which is feedback controlled [16, 20]. The FIC is preprogrammed and allows the eyes to quickly rotate inward or outward to the new visual target but does not always yield accurate movements. The FSC provides high accuracy. Since the FSC is feedback controlled, it requires time to guide the eyes to the desired target and reduce the error (difference between the eye’s current location and the desired target). One or both components may be modified with OBVAT. The main sequence can be used to quantitatively analyze the transient portion of disparity vergence step responses (also called jump ductions) where the FIC dominates the response [16, 18, 21-23]. Thus, analysis of changes in the main sequence after rehabilitation may help us understand whether significant changes are occurring in the FIC component of disparity vergence.

In a previous study [24], our laboratory studied 5 patients with symptomatic convergence insufficiency and concussion (mean age 22.2 years) and found a significant change in the main sequence ratio of convergence after OBVAT compared to the baseline measurements (P=0.05). Conversely, the main sequence ratio of divergence and saccades did not significantly change (P=0.1). Our laboratory has published other investigations showing optimization of the FIC in binocular normal controls after vergence therapy [25] and in those with CI [13, 26]. These results support the theory that one of the potential underlying mechanisms by which OBVAT leads to a sustained reduction in visual symptoms may be through the change in the neural control of the FIC.

The objective of this study is to confirm previous research and determine if there are significant changes in the disparity vergence main sequence of children with symptomatic convergence insufficiency after 12 weeks of OBVAT. This information may also help to establish the value of the main sequence ratio analysis as a useful method to understand the underlying mechanism(s) by which OBVAT improves disparity vergence and reduces visual symptoms in convergence insufficiency patients or other binocular vision disorders for future randomized clinical trials.

## Methods

The tenets of the Declaration of Helsinki were followed throughout the study. The institutional review board of Salus University approved the protocol and written informed consent and assent as well as Health Insurance Portability and Accountability Act (HIPAA) authorization were obtained before participation. The trial is registered with https://clinicaltrials.gov (Identifier: NCT03248336, date of registration 07/08/2017).

### Patient Selection

Patients were recruited from the Eye Institute of the Pennsylvania College of Optometry at Salus University and from the clinical practice of the one of the authors (MS). All participants had a comprehensive eye examination before the baseline visit. To be eligible, individuals had to be between 12 to 17 years old and have a diagnosis of symptomatic convergence insufficiency (CI). Symptomatic CI was defined as (1) a score of 16 or higher on the convergence insufficiency symptom survey (CISS); (2) exophoria at near at least 4 prism diopters (Δ) greater than at distance; (3) a receded near point of convergence of ≥ 6 cm break, and (4) insufficient positive fusional vergence (i.e., failing Sheard’s criterion or positive fusional vergence < 15Δ base-out) at near. Participants were required to have 20/25 visual acuity or better with best refractive correction if needed. Participants with a previous history of vision therapy or brain injury including concussion(s) were excluded. The participants also were required to have stable general health, intact cognitive function, and no other neurological conditions. The full eligibility criteria have been published previously (Scheiman, Talasan, Alvarez, 2019).

### Study Design

Clinical testing and objective eye movement recording were performed at baseline. After baseline measurements, participants received 12, one-hour visits of OBVAT. At the completion of therapy, all baseline testing was repeated.

### Clinical Testing

After obtaining written consent/assent, a vision examination was performed to determine if the patient was eligible for the study. Eligibility testing included administration of the Convergence Insufficiency Symptom Survey (CISS) to identify whether or not the patient was symptomatic [4, 27]. Other eligibility tests included best-corrected visual acuity at distance and near, a sensorimotor examination [cover test at distance and near, near point of convergence (20/30 target and the Gulden Near Point Rod), positive and negative fusional vergence at near (prism bar and a held 20/30 vertical line of letters as a target), vergence facility (12 base-out, 3 base-in prism) at distance and near, near stereoacuity (Randot Stereotest), monocular accommodative amplitude (20/30 target and the Gulden Near Point Rod), monocular accommodative facility (+/-2.00 flipper lenses and 20/30 vertical line of letters)], cycloplegic refraction, and an ocular health evaluation.

### Objective Outcome Measures of Disparity Vergence: Instrumentation

**Figure 1A and 1B:**
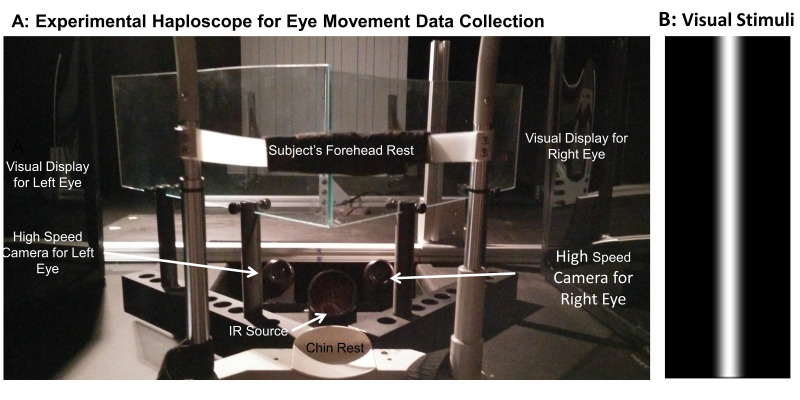
**Figure 1A:**Experimental set-up of haploscope used to record disparity vergence eye movements. **Figure 1B:**‘Difference of Gaussian’ (DoG) stimulus used for all vergence and saccadic testing.

The ISCAN RK-826PCI binocular tracking system (Burlington, MA, USA) recorded horizontal vergence eye movements at 240 frames per second (Figure 1A).

### Stimuli Presentation and Data Collection

To minimize blur cues and feedback and produce virtually accommodative ‘open loop’ conditions during the presentation of disparity vergence stimuli, a vertically oriented ‘difference of Gaussians’ (DoG) (Figure 1B) was used as the disparity vergence step stimuli within a traditional haploscope. The use of 4° (~7Δ) and 6° (~11Δ) symmetrical disparity steps were chosen to maximize the ability to gather quality data for each observation. Twelve observations of each of the visual stimuli were presented: 4° symmetrical disparity vergence steps from a vergence angle of 2° to 6°, 4° to 8°, 6° to 10°, 8° to 12° as well as 6° symmetrical disparity vergence steps from 6° to 12°, and from 4° to 10°. This resulted in a total of 72 convergent movements. The same stimuli were also used for divergence steps (72 divergent movements) for a total of 144 vergence movements. In addition, 40 responses each to 5° and 10° saccadic stimuli were recorded.

### Calibration

The participant’s head was restrained using a chin/head rest to minimize head movement and influence from the vestibular system [28]. A midline adjustment procedure was performed to insure proper positioning within the chin/head rest. Calibration consisted of a six-point, monocular calibration (1°, 3°, and 5° monocular, corresponding to 2°, 6°, 10° binocular vergence angle demand, or 4°, 5°, and 6° monocular, corresponding to 8°, 10°, 12° binocular vergence angle demand. Calibration for saccade responses consisted of a four-point, monocular calibration (5°, and 10° monocular into the left and right visual field). These calibrations were performed before and after completion of each eye movement response data collection.

### Eye Movement Analyses

Eye movement data were processed and analyzed with a custom MATLAB program (Waltham, MA, USA). All the 4° step vergence data were pooled for analysis, as were the 6° step vergence data because the outcome measures were not substantially different. The vergence eye movements were filtered with a 4^th^ order low pass Butterworth filter, with a cut off frequency of 40Hz to eliminate instrumentation noise especially 60Hz noise that is probably not physiological in nature. Saccadic eye movements were filtered with a 4^th^ order low pass Butterworth filter, with a cutoff frequency of 120Hz. Each individual left-eye and right-eye vergence movement response was manually inspected for the presence of any blink(s) or saccade(s) during any portion of the transient portion of the vergence eye movement. Saccades were easily identified because saccadic dynamics are an order of magnitude greater than vergence. Saccades that occurred during the transient portion of the vergence response were omitted from the peak velocity analysis because several studies suggest that saccades influence the maximum velocity of vergence [29-31]. Objective eye movement parameters assessed included, latency, time to peak velocity, peak velocity and response amplitude. Peak velocity was defined as the maximum value of the derivative within the transient portion of the vergence movement.

The main sequence ratio was used as the primary outcome measure for this study. The main sequence ratio is the peak velocity of the eye movement response divided by the response amplitude which is the amplitude the response attains within the transient portion of the movement. This technique has been used in many prior publications [13, 15-18]. The strength of the main sequence ratio is that it can be used to assess eye movement responses which do not have the range of movements as conducted by the original main sequence analysis (Bahill et al., 1975).

### Treatment: Office-based Vergence and Accommodative Therapy with Home Reinforcement (OBVAT)

Twelve, 60-minute, weekly visits of OBVAT were administered by a trained therapist combined with procedures to practice at home (15 minutes, 5 times per week). This treatment sequence is a well-accepted approach for treatment of convergence insufficiency [26] and has been successfully implemented in previous randomized clinical trials [7, 8]. Fifteen minutes of home-based therapy was prescribed to be performed 5 days per week, and compliance with home-based therapy was monitored at each visit by the therapist.

### Follow-up Visit

All participants were re-examined after completion of 12 weeks of OBVAT. Both the clinical and objective testing performed at enrollment were repeated at the outcome examination which occurred between 12-14 weeks after baseline.

### Statistical Analysis

All statistical analyses were performed using SPSS Version 24.0 with an alpha level of 0.05 used to determine statistical significance. For the main sequence analysis, we plotted the peak velocity as a function of response amplitude for convergence, divergence, and saccades for visual inspection to observe the distribution of the data. To determine whether significant differences were observed within all clinical and objective eye movement recording measurements, a two-tailed paired t-test was calculated. Effect size was determined using Cohen’s d effect size, using Cohen criteria of 0.2 = small effect, 0.5 = medium effect, and 0.8 = large effect, suggesting meaningful clinical significance.

## Results

Table 1 illustrates the changes that occurred in each clinical measure for the convergence insufficiency group from baseline to the outcome visit and show statistically significant (P<0.05) and clinically meaningful changes in all parameters except for the cover test at distance. Ten of the 12 participants (83%) in the convergence insufficiency group were categorized as “successful” based on pre-determined composite criteria (CISS, near point of convergence, positive fusional vergence), and the remaining two (participants 6 and 12) “improved”. Although these last two participants did not reach the required cutoff of <16 on the CISS, in both the CISS score decreased by 10 points or more (participant 6 by 19 points and participant 12 by 36 points). In addition, although participant 12 did not fully meet the criteria to be labeled “successful”, she did achieve the largest decrease in symptoms (36 points) of all the participants.

Tables 2 through 4 show the results of the objective eye movement testing which was performed at baseline and after 12 sessions (1 hour each) of OBVAT. In Table 2, the data show that for both 4° and 6° symmetrical convergence steps, there were statistically significant changes in peak velocity, time to peak velocity, and response amplitude. The data in Table 3 show statistically significant changes in peak velocity and response amplitude for 4° and 6° symmetrical divergence steps. Table 4 indicates that there were no statistically significant changes in saccadic eye movements from baseline to the outcome examination. All the statistically significant findings of mean change after OBVAT have a medium to large effect size (>0.5).

**Figure 2:**
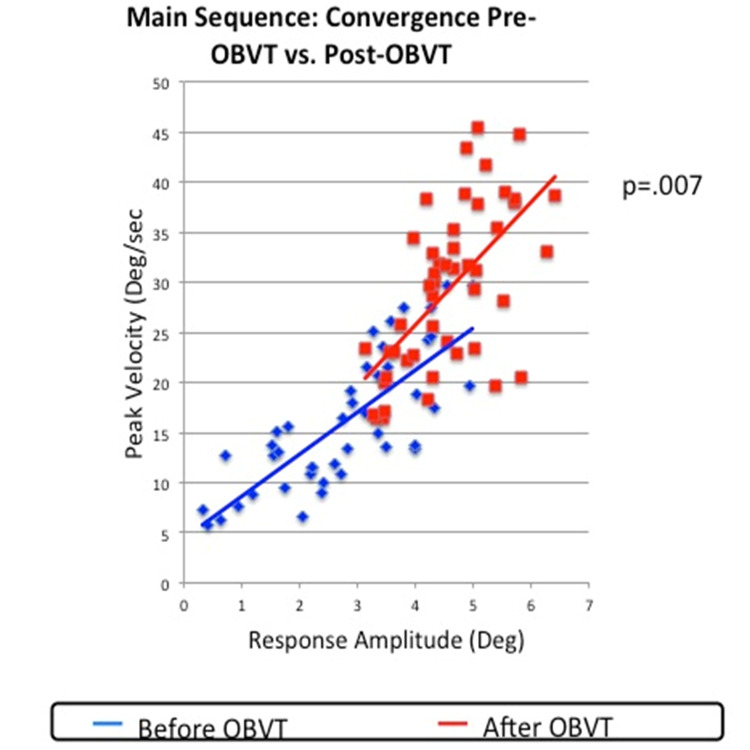
Main sequence for convergence pre- vs post-OBVAT

**Figure 3:**
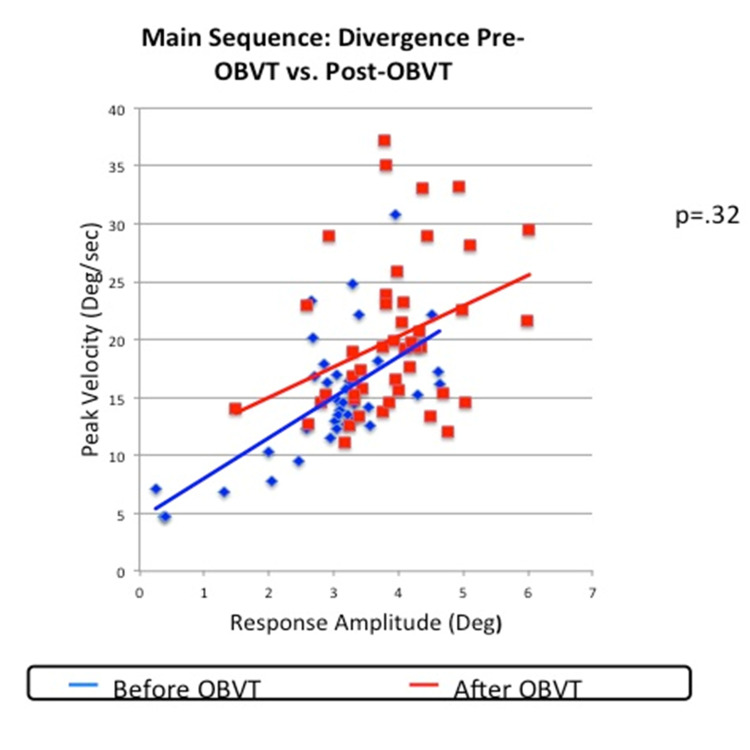
Main sequence for divergence pre- vs post-OBVAT

**Figure 4:**
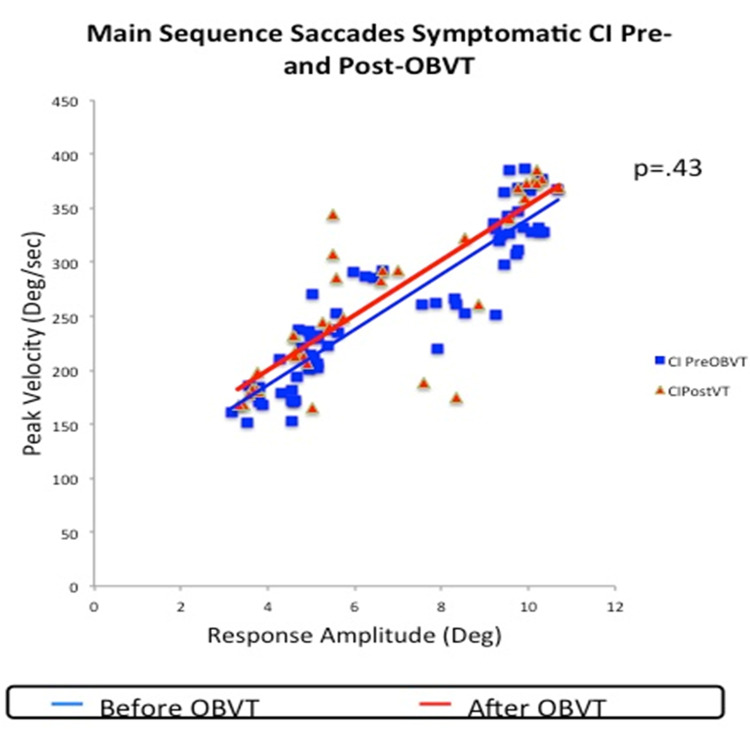
Main sequence for saccades pre- vs post-OBVAT

The main sequence plots for convergence (Figure 2), divergence (Figure 3) and saccades (Figure 4) report the P value for the change in the main sequence ratio for each type of eye movement comparing baseline to outcome measurements. A two-tailed paired t-test showed that a significant change in the main sequence ratio was observed for convergence after OBVAT compared to baseline measurements (P=0.007, Cohen’s d=0.5 medium effect). The average main sequence ratio (peak velocity divided by response amplitude) was 5.7 (sec^-1^) ± 1.5 before and changed to 6.2 (sec^-1^) ± 1.1 after OBVAT for convergence eye movements. Divergence and saccades did not exhibit significant changes in the main sequence ratio after OBVAT compared to the baseline measurements. Before OBVAT, the divergence main sequence ratio was 6.0 (sec^-1^) ± 4.4 and after OBVAT the main sequence ratio was 5.2 (sec^-1^) ± 1.8 (P=0.32). For saccades, the main sequence ratio was 38.9 (sec^-1^) ± 6.6 at baseline and 39.7 (sec^-1^) ± 11.6 after OBVAT (P=0.43).

**Table 1:** Comparison of Clinical Measures of Convergence Insufficiency Participants Pre-and Post-OBVAT

Function	Mean Pre-VT	Mean Post VT	Mean Change	Sig	Cohen’s *d* Effect Size	Confidence Interval
CISS	35.8	11.2	24.6	*P*=0.002	2.84	17.9 to 31.4
Cover Test (Distance) (Δ)*	-0.8	-0.5	0.3	*P*=0.16	0.16	-0.8 to 0.2
Cover Test (Near) (Δ)*	-9.2	-7.3	1.8	*P*=0.03	0.43	-3.2 to -0.4
Base-in Break (Δ)	14.8	19.6	4.8	*P*=0.02	0.74	-8.1 to -1.4
Base-out Break (Δ)	11.8	38.3	26.6	*P*=0.002	3.59	-32.9 to -20.2
Near point of convergence Break (cm)	16.5	3.6	12.9	*P*=0.002	4.03	10.2 to 15.6
Accommodation Amplitude Right Eye (cm)	14.8	8.5	6.3	*P*=0.003	1.94	3.7 to 8.9
Vergence Facility (FPM)	8.7	34.8	22.4	*P*=0.003	2.56	11.6 to 30.8
Monocular Accommodative Facility (FPM)	7.4	28.3	20.9	*P*=0.001	2.89	15.4 to 26.3
*(-) = exophoria						
FPM = Flips per minute						
Δ = prism diopter						

**Table 2:** Comparison of Objective Measures of Convergence Pre- and Post-OBVAT

Function	Mean Pre OBVAT	Mean Post OBVAT	Mean Change	Sig	Cohen’s *d* Effect Size	Confidence Interval
4° Symmetrical Convergence						
Peak Velocity (°/sec)	14.7	26.1	11.4	*P*=<0.001	1.48	8.3 to 15.0
Time to Peak Velocity(sec)	0.50	0.40	0.10	*P*=<0.001	0.99	.07 to .17
Response Amplitude (°)	2.6	4.1	1.5	*P*=<0.001	1.31	1.0 to 1.9
Latency (sec)	0.25	0.22	0.03	*P*=0.13	0.31	-01 to .07
6° Symmetrical Convergence						
Peak Velocity (°/sec)	14.7	26.1	11.4	*P*=<0.001	1.46	8.0 to 14.6
Time to Peak Velocity(sec)	0.50	0.40	0.10	*P*=0.01	0.51	0.05 to 0.15
Response Amplitude (°)	2.6	4.1	1.5	*P*=<0.001	1.15	1.0 to 1.9
Latency (sec)	0.23	0.25	0.02	*P*=0.31	0.12	-0.08 to 0.02

**Table 3:** Comparison of Objective Measures of Divergence Pre- and Post-OBVAT

Function	Mean Pre OBVAT	Mean Post OBVAT	Mean Change	Sig	Cohen’s *d* Effect Size	Confidence Interval
4° Symmetrical Divergence						
Peak Velocity (°/sec)	13.5	19.0	5.5	*P*=.01	0.62	1.4 to 9.6
Time to Peak Velocity(sec)	0.43	0.40	0.03	*P*=.25	0.27	-0.03 to 0.09
Response Amplitude (°)	2.7	3.6	0.9	*P*=.006	0.69	-0.3 to 1.5
Latency (sec)	0.21	0.23	0.02	*P*=0.46	0.16	-0.8 to 0.03
6° Symmetrical Divergence						
Peak Velocity (°/sec)	15.8	20.3	4.5	*P*=0.02	0.60	0.8 to 8.2
Time to Peak Velocity(sec)	0.53	0.56	0.03	*P*=0.28	0.26	0.05 to 0.15
Response Amplitude (°)	3.1	4.3	1.2	*P*=0.001	0.98	0.6 to 1.9
Latency (sec)	0.20	0.21	0.01	*P*=0.17	0.33	-0.03 to 0.01

**Table 4:** Comparison of Objective Measures of Saccades Pre- and Post-OBVAT

Function	Mean Pre OBVAT	Mean Post OBVAT	Mean Change	Sig	Cohen’s *d* Effect Size	Confidence Interval
5° Saccades						
Peak Velocity (°/sec)	202.5	212.8	10.3	*P*=.12	0.49	-23.7 to 3.1
Time to Peak Velocity(sec)	0.24	0.27	0.03	*P*=.35	0.28	-0.09 to 0.03
Response Amplitude (°)	4.6	4.7	0.1	*P*=.46	0.22	-0.4 to 0.2
Latency (sec)	0.23	0.22	0.01	*P*=.38	0.26	-0.01 to 0.03
10°Saccades						
Peak Velocity (°/sec)	312.1	312.0	0.1	*P*=0.99	0.003	-24.9 to 25.1
Time to Peak Velocity(sec)	0.23	0.23	0.01	*P*=0.38	0.27	0.01 to 0.03
Response Amplitude (°)	9.0	8.8	0.2	*P*=0.68	0.14	-0.4 to 0.6
Latency (sec)	0.21	0.21	0.01	*P*=0.55	0.18	-0.01 to 0.02

## Discussion

The results of this study are consistent with previous clinical trials [7, 8, 10, 12, 24] and show statistically significant and clinically meaningful changes in clinical and objective measures of vergence in participants with symptomatic convergence insufficiency treated with OBVAT for 12 weeks. The new contribution from this study is that statistically significant changes were also found for the main sequence ratio for convergence step responses, while there were no significant changes for either divergence or saccades responses post-OBVAT. This is not surprising since most of the therapy time was designed to improve convergence. The strength of a main sequence analysis which can be quantified as the main sequence ratio is it is an assessment of the underlying neural control of the responses. The main sequence analysis assesses whether the responses are simply scaled movements or whether the first-order dynamics are indeed different. The primary finding of this study is that the main sequence ratio of convergence significantly changed which supports a neural control change post OBVAT, whereas divergence and saccadic responses were not significantly different from a neural control aspect. Divergence peak velocity and response amplitude significantly changed but the main sequence ratio did not. Hence, the divergence responses were generated using similar neural control and post OBVAT the divergence responses were larger in magnitude and speed. Conversely, not only did convergence movement increase in speed and magnitude, the main sequence ratio or the neural control strategy was significantly different post-OBVAT.

Neurophysiological studies support that ‘velocity-encoding’ burst cells are observed within the oculomotor nucleus within the brainstem [32]. These cells are responsible for the bursting activity to move the eyes quickly to the visual target but do not necessarily do so very accurately [33, 34]. Adaptive changes corresponding to modifications in peak velocity and vergence response amplitudes is modeled as an improvement to the fusional-initiating component. These data of the present study report significant changes in peak velocity and the main sequence ratio. Hence, these data support that one of the underlying physiological changes that occur after OBVAT is optimization of the fusion-initiating component of convergence.

Vergence therapy can generally be performed two ways [2]. The first method is using a ramp or tonic vergence stimulus. In a ramp/tonic vergence procedure, the vergence demand is increased in a gradual manner. The second method is phasic or step vergence therapy. In a phasic/step procedure, the vergence demand is changed in discrete steps and speed of the response is emphasized. Some research has been done comparing the effectiveness of these two types of procedures [35]. This evidence suggests that both procedures are effective in producing gains in fusional vergence, although phasic/step vergence therapy shows the greatest improvements [35]. Typically, both types of procedures are utilized with a patient undergoing OBVAT. Given that the main sequence ratio is a surrogate for the fusion-initiating component described within the Dual Mode model of disparity vergence, the results of this study emphasize the importance of phasic/step vergence when planning a OBVAT program for convergence insufficiency [36]. The objective of this type of therapy is to improve the dynamics of the vergence response which may optimize the fusion-initiating component. When the fusion-initiating component is enhanced, meaning it brings the eyes closer to the intended visual stimulus, less adjustment is needed by the fusion-sustaining component.

While OBVAT has been shown to be an effective treatment for convergence insufficiency [6-11] and accommodative disorders [37], these studies show about 25% of participants do not achieve a successful outcome after this treatment. Thus, there is a need to better understand the underlying mechanisms that explain the improvement in vision function after vison therapy. Such information will help clinicians understand how to modify OBVAT protocols to improve success rates.

There are a few study limitations. The lack of either a placebo control group or an untreated control group prevents us from definitively ruling out other potential reasons for improvement in the outcome data (such as the placebo effect, Hawthorne effects, regression to the mean, etc.). However, the significant differential effects on convergence compared to no significant change on divergence or saccadic responses in terms of the neural control strategy as assessed by the main sequence ratio does support that OBVAT changed the convergence system in a different manner compared to the divergence or saccadic systems. Previous studies [7-11, 25] in which a placebo control group was used, suggest that the observed effects were treatment-related. Another potential issue is whether the repetitions of vergence movements during the assessment could have changed the vergence dynamics. There are studies showing, that repetition during an assessment changes transient vergence dynamics [38, 39]. However, the same effect of repetition was present at baseline and outcome and likely cancelled any effect on the actual mean change. Finally, the protocol used to objectively assess disparity only included step vergence stimuli and did not include ramp stimuli which can provide constant stimulation for the fusion-sustaining component. Thus, the stimulus used within this study concentrates on the fusion-initiating component. In future studies, the design should also incorporate ramp stimuli of slower speeds which prior study support studies the fusion sustaining component [15, 40-43]. Ultimately, it will be necessary to perform several clinical trials comparing the effectiveness of therapy designed to treat the two components described within the dual-mode theory.

Future research may wish to include a phoria adaptation and fixation disparity analysis at baseline and as an outcome measure for OBVAT to assess changes to the slow fusion system of vergence [44] and the error within the disparity vergence system [45]. Neither of these were investigated within this present study and would facilitate a broader understanding of how OBVAT influences the vergence system.

### Ethics and Conflict of Interest

The author(s) declare(s) that the contents of the article are in agreement with the ethics described by the Journal and that there is no conflict of interest regarding the publication of this paper.

### Acknowledgements

This research was supported by the National Eye Institute of the National Institutes of Health NEI R01EY023261 to TLA, Department of Health and Human Services, Bethesda, Maryland, USA and National Science Foundation NSF MRI CBET 1428425.
